# Ni‐CeO_2_ Heterostructures in Li‐S Batteries: A Balancing Act between Adsorption and Catalytic Conversion of Polysulfide

**DOI:** 10.1002/advs.202105538

**Published:** 2022-04-12

**Authors:** Yang Kong, Xin Ao, Xiao Huang, Jinglong Bai, Shangquan Zhao, Jinyong Zhang, Bingbing Tian

**Affiliations:** ^1^ School of Material and Physics China University of Mining and Technology Xuzhou Jiangsu 221008 China; ^2^ SZU‐NUS Collaborative Innovation Center for Optoelectronic Science and Technology International Collaborative Laboratory of 2D Materials for Optoelectronics Science and Technology of Ministry of Education Institute of Microscale Optoelectronics Shenzhen University Shenzhen 518060 China; ^3^ School of Materials Science and Engineering Nanchang University 999 Xuefu Avenue Nanchang Jiangxi 330031 China

**Keywords:** adsorption of polysulfide, catalytic conversion, Li–S batteries, Ni‐CeO_2_ heterostructures

## Abstract

Lithium–sulfur (Li–S) batteries have attracted considerable attention over the last two decades because of a high energy density and low cost. However, the wide application of Li–S batteries has been severely impeded due to the poor electrical conductivity of S, shuttling effect of soluble lithium polysulfides (LiPSs), and sluggish redox kinetics of S species, especially under high S loading. To address all these issues, a Ni–CeO_2_ heterostructure‐doped carbon nanofiber (Ni‐CeO_2_‐CNF) is developed as an S host that combines the strong adsorption with the high catalytic activity and the good electrical conductivity, where the LiPSs anchored on the heterostructure surface can directly gain electrons from the current collector and realize a fast conversion between S_8_ and Li_2_S. Therefore, Li–S batteries with S@Ni‐CeO_2_‐CNF cathodes exhibit superior long‐term cycling stability, with a capacity decay of 0.046% per cycle over 1000 cycles, even at 2 C. Noteworthy, under a sulfur loading up to 6 mg cm^−2^, a high reversible areal capacity of 5.3 mAh cm^−2^ can be achieved after 50 cycles at 0.1 C. The heterostructure‐modified S cathode effectively reconciles the thermodynamic and kinetic characteristics of LiPSs for adsorption and conversion, furthering the development of high‐performance Li–S batteries.

## Graphical abstract

Experimental measurements and theoretical calculations show that the Ni‐CeO_2_ heterostructure combines the strong adoption of LiPSs with excellent catalytic activity for accelerating the redox kinetics between S_8_ and Li_2_S. The present study provides an effective strategy for accommodating the thermodynamic and kinetic characteristics of LiPSs adsorption and conversion.The material prepared in this study has considerable application potential in high‐performance Li‐S batteries.

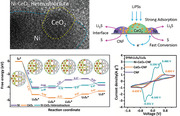

## 1 Introduction

Lithium–sulfur (Li–S) batteries are considered ideal candidates for next‐generation energy storage devices due to a high theoretical specific capacity (1675 mAh g^−1^) and energy density (2600 Wh kg^−1^).^[^ ^1^ ^]^ Furthermore, interest in Li–S batteries has been increasing because of the low cost, natural abundance, nontoxicity, and environmental friendliness of sulfur (S).^[^ ^2^ ^]^ However, commercial application of these batteries has been severely hindered by several intrinsic characteristics, that is, the insulating property of sulfur and its discharge product Li_2_S,^[^ ^3^ ^]^ large volume expansion (≈80%) from S_8_ to Li_2_S during the discharge process,^[^ ^4^ ^]^ sluggish redox kinetics of the reduction of S_8_ or oxidation of Li_2_S,^[^ ^5^ ^]^ and the shuttle effect of soluble lithium polysulfides (LiPSs, Li_2_S*_n_*_,_ 2 ≤ *n* ≤ 8) intermediates,^[^ ^6^ ^]^ leading to the low utilization of sulfur active materials and consequently poor cycling performance of Li–S batteries.

To date, strenuous efforts have been made to address the abovementioned issues.^[^ ^7^ ^]^ Various carbon materials (e.g., carbon nanotubes,^[^ ^8^ ^]^ graphene,^[^ ^9^ ^]^ porous carbon,^[^ ^10^ ^]^ and carbon nanofibers^[^ ^11^ ^]^) have been widely used as support materials to improve the electrical conductivity of S cathodes and confine S.^[^ ^12^, ^13^, ^14^ ^]^ However, the weak physical interaction between nonpolar carbon materials and LiPSs results in ineffective suppression of the shuttle effect of LiPSs by carbon materials during electrochemical cycling, which cause LiPSs to dissolve in the electrolyte.^[^ ^12^, ^15^ ^]^ To enhance chemical adsorption with LiPSs, polar materials (including various metal particles,^[^ ^16^, ^17^ ^]^ oxides,^[^ ^18^, ^19^, ^20^ ^]^ sulfides,^[^ ^14^, ^21^ ^]^ nitrides,^[^ ^22^ ^]^ and carbides^[^ ^23^ ^]^) have been mixed/doped with carbon materials as sulfur hosts for Li–S batteries. Experimental and theoretical studies have shown that polar oxides can effectively immobilize LiPSs by strong chemisorption.^[^ ^24^ ^]^ Many polar oxides, such as SiO_2_,^[^ ^12^ ^]^ SnO_2_^[^ ^18^ ^]^ MnO_2_,^[^ ^25^ ^]^ CeO_2_,^[^ ^26^, ^27^ ^]^ TiO_2_,^[^ ^19^, ^28^ ^]^ MgO,^[^ ^29^ ^]^ and VO_2_,^[^ ^30^ ^]^ have been introduced into cathodes to effectively adsorb LiPSs by chemical interactions. However, most of the metal oxides have poor electrical conductivities and cannot directly gain electrons from a current collector, such that the LiPSs anchored on the carbon surface cannot fully participate in electrochemical reactions. Thus, metal oxides are generally composed/doped with carbon materials as S carriers for cathodes in Li–S batteries. LiPSs can thus diffuse to the conductive carbon surface to complete the reaction.^[^ ^14^, ^28^, ^29^, ^30^, ^31^ ^]^ Unfortunately, carbon materials exhibit weak catalytic activity in accelerating the redox kinetics of S_8_ and Li_2_S. The strong adsorption and weak conversion of LiPSs results in insufficient utilization of S and poor cycling performance. Transition metals, such as Fe,^[^ ^32^ ^]^ Co,^[^ ^33^ ^]^ and Ni,^[^ ^16^, ^34^ ^]^ can provide the high electrical conductivity and good catalytic conversion in Li–S batteries, but the nonpolar metal particles cannot effectively anchor LiPSs and suppress the shuttle effect during cycling.

**Figure 1:**
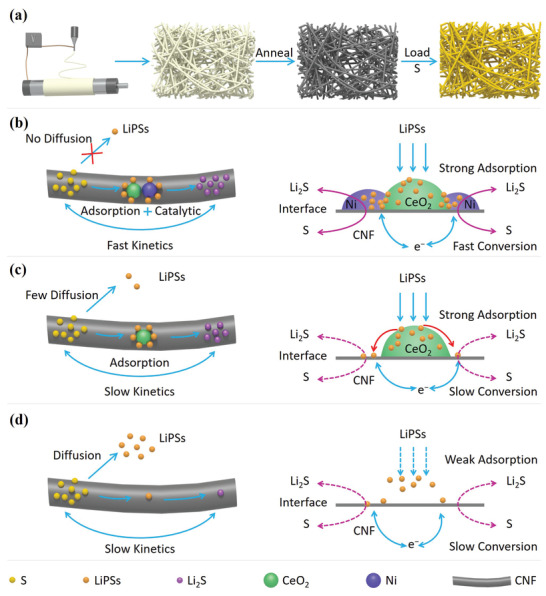
a) Schematic of the synthesis of S@Ni‐CeO_2_‐CNF and the operational principles of b) Ni‐CeO_2_‐CNF, c) CeO_2_‐CNF, and d) CNF in Li‐S batteries.

In this work, we innovatively present a Ni‐CeO_2_ heterostructure‐doped carbon nanofiber (Ni‐CeO_2_‐CNF) as an S host material for Li–S batteries (**Figure** **1**a). Experimental measurements and theoretical calculations show that the Ni–CeO_2_ heterostructure combines the strong adsorption with excellent catalytic activity and good electric conductivity, where the LiPSs anchored on the heterostructure can directly gain electrons from the current collector and realize a fast conversion between S_8_ and Li_2_S (Figure 1b). In the absence of catalysts, the S cathode with CeO_2_‐doped carbon nanofibers (S@CeO_2_‐CNF) has strong adsorption for LiPSs but achieves slow conversion of LiPSs (Figure 1c). By sharp contrast, carbon nanofibers (CNFs) without CeO_2_ and Ni catalysts form a long‐range conductive network but have weak adsorption and achieve slow conversion of LiPSs, leading to poor cycling performance (Figure 1d). As a result, in the presence of Ni‐CeO_2_‐CNF, the sulfur cathode exhibits both excellent long‐term cycling stability (a low decay rate of 0.046% per cycle during 1000 cycles at 2 C) and a high‐rate capability (553.8 mAh g^−1^ even at a 3 C rate). Meanwhile, under a sulfur loading up to 6 mg cm^−2^, a high reversible areal capacity of 5.3 mAh cm^−2^ can be reached after 50 cycles at 0.1 C. Thus, strong adsorption and fast redox kinetics of LiPSs are regulated in S cathodes, promoting the practical application of Li–S batteries.

## 2 Results and Discussions

**Figure 2:**
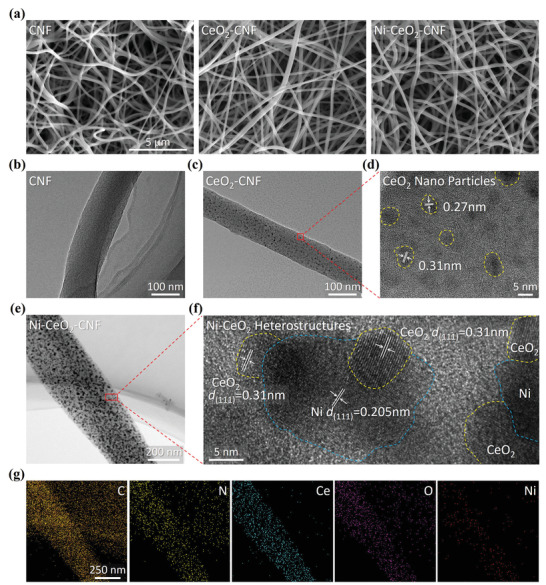
SEM images of a) CNF, CeO_2_‐CNF, and Ni‐CeO_2_‐CNF; TEM images of b) CNF, c) CeO_2_‐CNF, and e) Ni‐CeO_2_‐CNF; HR‐TEM images of d) CeO_2_‐CNF, and f) Ni‐CeO_2_‐CNF; g) selected area and the corresponding element mappings of Ni‐CeO_2_‐CNF.

SEM images of CNFs, CeO_2_‐CNFs, and Ni‐CeO_2_‐CNFs obtained after high‐temperature annealing (900 °C) present a crosslinked nanofiber morphology, where each fiber appears virtually uniform with a diameter of ≈200 nm (**Figure** **2**a). A TEM image of CNFs (Figure 2b) shows no nanoparticles on the surface. By contrast, abundant nanoparticles uniformly decorate the CeO_2_‐CNF and Ni‐CeO_2_‐CNF surfaces (Figure 2c,e). Clear lattice fringes of CeO_2_ can be observed in a high‐resolution transmission electron microscopy (HR‐TEM) image of CeO_2_‐CNF (Figure 2d). In particular, an HR‐TEM image (Figure 2f) of Ni‐CeO_2_‐CNF exhibits clear lattice fringes of Ni(111) and CeO_2_ (111), demonstrating the in situ formation of Ni‐CeO_2_ heterostructures.^[^ ^35^ ^]^ The Ni‐CeO_2_ heterostructures with homogeneous distributions of Ni, Ce and O elements are further confirmed by EDS elemental mappings (Figure 2g).

**Figure 3:**
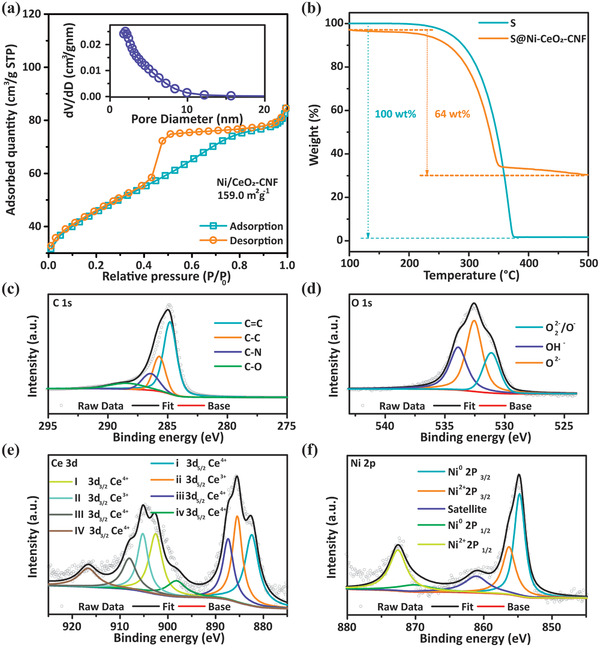
a) N_2_ adsorption‐desorption isotherms of Ni‐CeO_2_‐CNF (the inset shows the pore size distribution); b) TGA curves of S and S@Ni‐CeO_2_‐CNF; XPS spectra of c) C 1 s, d) O 1 s, e) Ce 3d, and f) Ni 2p of Ni‐CeO_2_‐CNF.

X‐ray diffraction (XRD) patterns (Figure S4, Supporting Information) of CNF, CeO_2_‐CNF, and Ni‐CeO_2_‐CNF exhibit relatively intense peaks at 25.5° (JCPDS 26–1076) and 44.5° (JCPDS 50–1086), which are in good agreement with those of graphitized carbon materials.^[^ ^36^ ^]^ To further evaluate the graphitic degree of the samples, Raman spectra were analyzed (Figure S5, Supporting Information). There are two typical characteristic peaks at 1350 cm^−1^ (D band) and 1580 cm^−1^ (G band) of disordered carbon and ordered graphitic carbon, respectively.^[^ ^9^ ^]^ The intensity ratios of the D‐to‐G band (*I*_D_/*I*_G_), representing the carbon graphitization degree of CNFs, CeO_2_‐CNFs, and Ni‐CeO_2_‐CNFs, are all close to 1, indicating similar graphitization of the three materials. The specific surface area and pore structure of CNF (Figure S6, Supporting Information), CeO_2_‐CNF (Figure S6, Supporting Information) and Ni‐CeO_2_‐CNF (**Figure** **3**a) were characterized using nitrogen adsorption‐desorption isotherms.^[^ ^26^, ^37^ ^]^ The specific surface areas of CNF, CeO_2_‐CNF, and Ni‐CeO_2_‐CNF are 66.7, 232.4, and 159.0 m^2^ g^−1^, respectively. The pore volumes of CNF, CeO_2_‐CNF, and Ni‐CeO_2_‐CNF are 0.05, 0.14, and 0.13 cm^3^ g^−1^, respectively. The BJH pore size distribution curves demonstrate the presence of mesopores in the material structures (2–10 nm). Meanwhile, CNF and CeO_2_‐CNF presence a small number of micropores, as confirmed by the BJH pore size distribution curves. TGA measurements were used to determine the sulfur contents of the S@CeO_2_‐CNF and S@Ni‐CeO_2_‐CNF composites. The weight content of S in the S@CeO_2_‐CNF and S@Ni‐CeO_2_‐CNF were determined to be 64 wt% from the change in the respective curve (Figure S7, Supporting Information, Figure 3b). The chemical bonding states of Ni‐CeO_2_‐CNF were studied by X‐ray photoelectron spectroscopy (XPS). The C 1s spectrum of Ni‐CeO_2_‐CNF can be fit with four peaks (Figure 3c) corresponding to C═C (284.8 eV), C—C (285.7 eV), C—N (286.5 eV) and C—O (288.5 eV).^[^ ^8^ ^]^ The C—N peak was produced by the decomposition of PAN and PVP. Figure 3d shows the XPS spectra of O 1 s, in which the peaks at 531.1, 532.5, and 533.8 eV are attributed to lattice oxygen (O^2−^), adsorbed oxygen species (O_2_^2−^), and hydroxyl species (OH^−^), respectively.^[^ ^38^ ^]^ The XPS spectra (Figure 3e) exhibits Ce 3d oxidation states at 885.6 and 903.5 eV that are assigned to Ce 3d_5/2_ and Ce 3d_3/2_, respectively. The ii and II peaks are assigned to Ce^3+^. The i, iii, iv, I, III, and IV peaks are related to Ce^4+^.^[^ ^38^, ^39^ ^]^ The high‐resolution XPS spectra (Figure 3f) of Ni 2p in Ni‐CeO_2_‐CNF can be fitted to the zero‐valence state (854.5 and 870.1 eV) and ionic state (856.5 and 872.5 eV) with satellites from the Ni—O species (861.1 eV), verifying the existence of metallic Ni. The C 1s, Ce 3d and Ni 2p (Figure S8, Supporting Information) of S@Ni‐CeO_2_‐CNF were further analyzed. The results show that Ni‐CeO_2_‐CNF loaded with S is stable.

**Figure 4:**
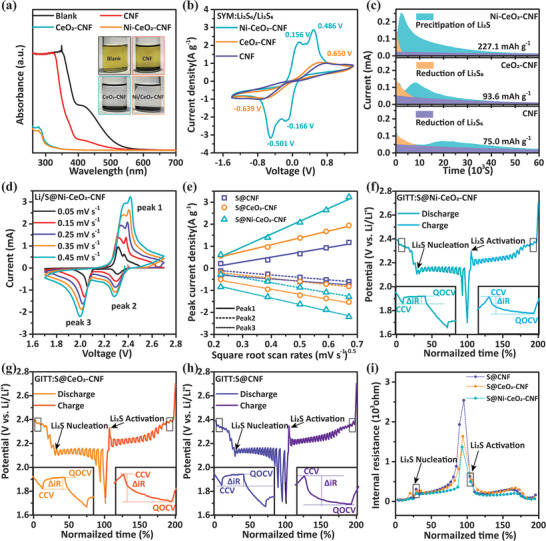
a) UV–vis spectra and optical images of CNF, CeO_2_‐CNF, and Ni‐CeO_2_‐CNF with adsorbed LiPS solutions; b) CV profiles of CNF, CeO_2_‐CNF, and Ni‐CeO_2_‐CNF symmetric cells; c) potentiostatic discharge profile at 2.05 V for different electrodes with a Li_2_S_8_ catholyte to evaluate the nucleation kinetics of Li_2_S; d) CV curves of the S@Ni‐CeO_2_‐CNF electrode at various scanning rates; e) profiles of CV peak currents versus the square root of the scan rates; galvanostatic intermittent titration (GITT) voltage profiles of f) S@Ni‐CeO_2_‐CNF, g) S@CeO_2_‐CNF, and h) S@CNF cathode at 0.1 C; i) internal resistances of the S@Ni‐CeO_2_‐CNF, S@CeO_2_‐CNF, and S@CNF electrodes relative to the normalized discharge–charge time.

To corroborate the chemical interaction between the host matrix and LiPSs, a polysulfide adsorption visualization test was conducted on CNF, CeO_2_‐CNF, and Ni‐CeO_2_‐CNF. The addition of CeO_2_‐CNF and Ni‐CeO_2_‐CNF to a Li_2_S_6_ solution causes the solution color to change from dark yellow to colorless (transparent, inset of **Figure** **4**a). Soluble Li_2_S_6_ is almost completely adsorbed by CeO_2_‐CNF and Ni‐CeO_2_‐CNF, as confirmed by UV–vis results, in which the characteristic peaks of Li_2_S_6_ (280 nm) and Li_2_S_4_ (410 nm) are absent (Figure 4a).^[^ ^40^ ^]^ By contrast, the solution containing CNFs and the blank Li_2_S_6_ solution remain yellow, and distinct characteristic peaks of Li_2_S_6_ and Li_2_S_4_ appear in the UV–vis spectra (Figure 4a). All the above mentioned results indicate that the CeO_2_‐CNF and Ni‐CeO_2_‐CNF composite powders have a strong adsorption ability for soluble LiPSs. To determine the catalytic activity of Ni‐CeO_2_‐CNF toward the reduction and oxidation of LiPSs, cyclic voltammetry (CV) measurements were performed on symmetrical cells with CNF, CeO_2_‐CNF, and Ni‐CeO_2_‐CNF as the working and counter electrodes. In Figure 4b, the CV curves of Ni‐CeO_2_‐CNF show four pronounced redox peaks and significantly higher current‐density peaks than those of CNF and CeO_2_‐CNF, implying superior electrocatalytic activity for S reaction kinetics.^[^ ^41^ ^]^ To analyze the superiority of Ni‐CeO_2_‐CNF toward Li_2_S nucleation, potentiostatic discharge curves (Figure 4c) of CNF, CeO_2_‐CNF, and Ni‐CeO_2_‐CNF cells were tested at 2.05 V with an Li_2_S_8_ solution as the electrolyte. The Ni‐CeO_2_‐CNF electrode exhibits the earliest and highest current responses (0.252 mA at 2170 s for Ni‐CeO_2_‐CNF, 0.127 mA at 8185 s for CeO_2_‐CNF, and 0.068 mA at 20360 s for CNF). The Ni‐CeO_2_‐CNF electrode also exhibits a higher Li_2_S precipitation capacity (227.1 mAh g^−1^) than the CeO_2_‐CNF (93.6 mAh g^−1^) and CNF electrodes (75.0 mAh g^−1^). This result indicates a lower barrier to Li_2_S formation on the Ni‐CeO_2_ heterostructures compared to CeO_2_‐CNF and CNF.^[^ ^42^ ^]^ The diffusivity of Li ions was determined from the CV curves of S@CNF (Figure S9), S@CeO_2_‐CNF (Figure S10, Supporting Information) and S@Ni‐CeO_2_‐CNF (Figure 4d) cathodes assembled in Li–S batteries. The linear relationship between the peak current density (*I*_peak_) and the square root scanning rate (*v* ^0.5^) is expressed by the Randles–Sevcik equation: *I*_p_ = (2.65 × 10^5^)*n* ^1.5^ *SD*_Li+_^0.5^Δ*C*_Li+_ *v* ^0.5^, where *n* denotes the number of transfer charges, S denotes the surface area of the active electrode, *D*_Li+_ denotes the diffusion coefficient of Li ions, and Δ*C*_Li+_ denotes the concentration of Li ions.^[^ ^43^ ^]^ The peak currents at the square root of different scan rates are shown in Figure 4e. The slopes of the S@CNF, S@CeO_2_‐CNF, and S@Ni‐CeO_2_‐CNF curves are 2.18, 3.11, and 5.83, respectively, at peak 1; 1.10, 1.40, and 2.28, respectively, at peak 2; and 0.98, 2.27, and 2.98, respectively, at peak 3. The significantly larger slope corresponding to the S@Ni‐CeO_2_‐CNF compared to those of the other cathodes demonstrates a higher diffusion rate of Li ions and faster kinetics of LiPSs conversion during the redox process. To further investigate the internal resistances of cells with S@CNF, S@CeO_2_‐CNF, and S@Ni‐CeO_2_‐CNF cathodes during the discharge/charge process, galvanostatic intermittent titration (GITT) profiles were measured at 0.1 C. The internal resistances to nucleation and activation of Li_2_S are indicated by the dip depth in the discharging and charging profiles (shown by the arrows in Figure 4f–h).Polarization during the discharge−charge process can be quantified by the relative size of Δ*R*_internal_ in GITT tests according to the following relation:

(1) $$\Delta R_{\mathrm{internal}}\left(\Omega \right)=\left|\Delta V_{\mathrm{QOCV}-\mathrm{CCV}}\right|/I_{\mathrm{applied}}$$

where Δ*V*_QOCV–CCV_ is the voltage difference between the points of quasi open‐circuit voltage (QOCV) and closed‐circuit voltage (CCV), and *I*_applied_ is the applied current, as shown in Figure 4f–h inset. The S@Ni‐CeO_2_‐CNF battery exhibits smaller Δ*R*_internal_ values than the other batteries (Figure 4i) between the Li_2_S nucleation and activation points, indicating that the S@Ni‐CeO_2_‐CNF electrodes have the lowest internal resistance. All the results of the abovementioned experiments indicate that the Ni‐CeO_2_ heterostructures have a strong adsorption ability for soluble LiPSs and effectively accelerate the electrochemical reaction kinetics during the redox process.

**Figure 5:**
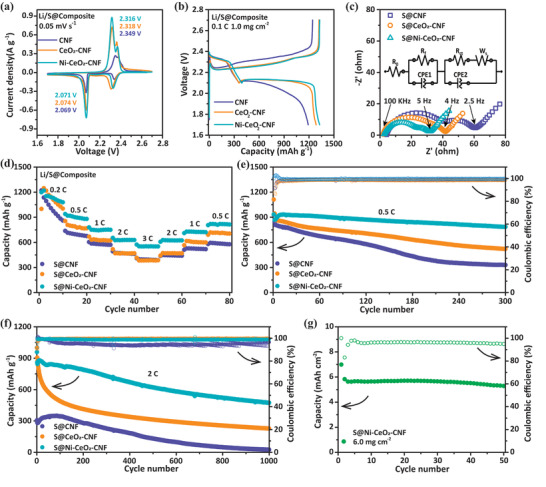
Electrochemical performances of the S@CNF, S@CeO_2_‐CNF, and S@Ni‐CeO_2_‐CNF cathodes: a) CV profiles; b) galvanostatic discharge/charge profiles for the first cycle at 0.1 C; c) EIS curves; d) rate performances and cycling performance at e) 0.5 C and f) 2 C of S@CNF, S@CeO_2_‐CNF, and S@Ni‐CeO_2_‐CNF cathodes; g) cycling performance of S@Ni‐CeO_2_‐CNF cathode at 0.1 C with high S loadings of 6.0 mg cm^−2^.

The CV profiles of the cells with S@Ni‐CeO_2_‐CNF between 1.7 and 2.7 V at a scan rate of 0.05 mV s^−1^ are clearly shown in **Figure** **5**a. The two cathodic peaks at 2.328 and 2.071 V correspond to the reduction of S_8_ to high‐order LiPSs and further reduction of LiPSs to short‐chain solid Li_2_S_2_/Li_2_S. The two anodic peaks are attributed to the reverse process of transforming solid Li_2_S_2_/Li_2_S to LiPSs (2.316 V) and further to S_8_ (2.369 V).^[^ ^44^ ^]^ Compared to S@CNF and S@CeO_2_‐CNF (Figure 5a), S@Ni‐CeO_2_‐CNF has a cathodic peak at a higher potential (2.071 V) and anodic peaks at lower potentials (2.316 V), indicating enhanced reaction kinetics. Good overlap of the initial three CV cycles also suggests good electrochemical reversibility for the cell with S@Ni‐CeO_2_‐CNF (Figure S11, Supporting Information).

The galvanostatic discharge/charge profiles of batteries with different materials at 0.1 C are compared in Figure 5b. The initial discharge capacity is 1327.6 mAh g^−1^ for S@Ni‐CeO_2_‐CNF, 1286.2 mAh g^−1^ for S@CeO_2_‐CNF, and 1190.2 mA h g^−1^ for S@CNF. The discharge/charge plateaus are significantly longer for S@Ni‐CeO_2_‐CNF than the other materials, resulting in a higher capacity. Notably, the cell with the S@Ni‐CeO_2_‐CNF cathode shows a stable and flat discharge plateau with the lowest polarization potential of 0.116 V. The electrochemical impedance spectroscopy (EIS) curves of the different batteries after ten cycles are shown in Figure 5c. The semicircle in the high‐frequency region reflects the charge‐transfer resistance at the carbon interface, and the semicircle in the medium‐frequency region indicates the formation of a solid Li_2_S@Li_2_S_2_ film on the carbon interface.^[^ ^45^ ^]^ The cell with S@Ni‐CeO_2_‐CNF produces the smallest semicircle in the Nyquist plot, indicating high transportation of ions and more ideal conductivity, which can also decrease the charge‐transfer resistance.

The cells with S@Ni‐CeO_2_‐CNF also display the highest rate performance for rates ranging from 0.2 to 3 C (Figure 5d). The discharge capacity of S@Ni‐CeO_2_‐CNF reaches 1197.9 (0.2 C), 938.6 (0.5 C), 779.1 (1 C), 653.2 (2 C), and 572.8 mAh g^−1^ (3 C). An average specific capacity of 553.8 mAh g^−1^ can be achieved even at a high rate of 3 C. As the current rate is returned from 3 to 0.5 C, S@Ni‐CeO_2_‐CNF retains a high average specific capacity of 813.6 mAh g^−1^. This result indicates that the Li–S batteries with S@Ni‐CeO_2_‐CNF have a good rate capability and cycle stability. In Figure S12, Supporting Information, the capacity of the S@Ni‐CeO_2_‐CNF cathode is initially 1208.7 mAh g^−1^ and maintained at 919.5 mAh g^−1^ after 100 cycles at 0.2 C. The capacity of the S@Ni‐CeO_2_‐CNF cathode is initially 933.4 mAh g^−1^ and maintained at 783.6 mAh g^−1^ after 300 cycles at 0.5 C (Figure 5e). By contrast, the capacities of the S@CNF and S@CeO_2_‐CNF cathodes decay faster (329.0 and 520.8 mAh g^−1^, respectively) after 300 cycles. Moreover, the S@Ni‐CeO_2_‐CNF cathode exhibits superior cycling performance with a small capacity decay of 0.046% per cycle during 1000 cycles at 2 C (Figure 5f). To further explore the electrochemical performance of S@Ni‐CeO_2_‐CNF cathodes, a series of tests were carried out under high S loadings of 3.0, 4.0, and 6.0 mg cm^−2^. A high areal capacities of 5.3 mAh cm^−2^ (0.1 C) after 50 cycles can be obtained under a sulfur loading of 6 mg cm^−2^ (Figure 5g). When S loadings are 3.0 and 4.0 mg cm^−2^, the initial specific capacities at 0.1 C after the activation process are 1116.4 and 1091.8 mAh g^−1^, respectively, and the remaining capacities after 80 cycles are 774.1 and 683.8 mAh g^−1^ (Figure S13, Supporting Information). Therefore, these results illustrate the good electrochemical performances of the S@Ni‐CeO_2_‐CNF cathode even under a high S loading.

**Figure 6:**
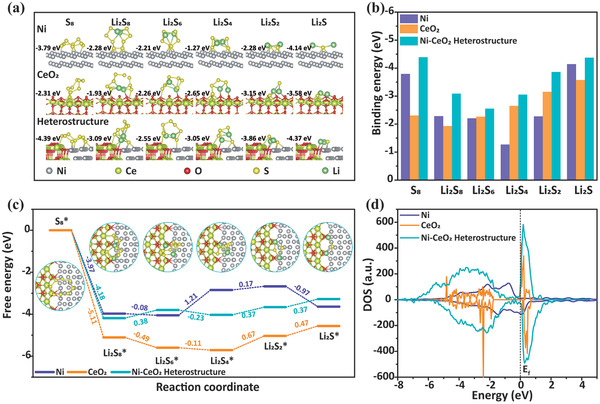
a) Optimized binding geometric configurations and energies of Ni, CeO_2_ and Ni‐CeO_2_ heterostructures with S_8_, Li_2_S_8_, Li_2_S_6_, Li_2_S_4_, Li_2_S_2_, and Li_2_S; b) binding energies of Ni, CeO_2_, and Ni‐CeO_2_ heterostructures with S_8_, Li_2_S_8_, Li_2_S_6_, Li_2_S_4_, Li_2_S_2_, and Li_2_S calculated using DFT; c) energy profiles for the discharging process from S_8_ to Li_2_S on the Ni, CeO_2_, and Ni‐CeO_2_ heterostructure models (insets show the optimized adsorption conformations of intermediate species on the Ni‐CeO_2_ heterostructure models); d) total density of states for the Ni and Ni‐CeO_2_ heterostructures.

**Figure** **6**a,b show the binding energies of Ni, CeO_2_, and Ni‐CeO_2_ heterostructures with S_8_, Li_2_S_8_, Li_2_S_6_, Li_2_S_4_, Li_2_S_2_, and Li_2_S. The Ni‐CeO_2_ heterostructure exhibits the stronger adsorption ability for soluble LiPSs than Ni and CeO_2_. Therefore, the Ni‐CeO_2_ heterostructure can efficiently mitigate the shuttling of LiPSs.^[^ ^32^, ^46^ ^]^ To explain the improved conversion of LiPSs by the S@Ni‐CeO_2_‐CNF cathode, we performed DFT calculations for the Gibbs free energies of different possible reactions of LiPSs on the Ni‐CeO_2_ heterostructure and compared the results to those for similar reactions on Ni and CeO_2_. During the discharge process, S_8_ undergoes double reduction with two Li^+^ ions to form Li_2_S_8_. Subsequently, Li_2_S_8_ undergoes further reduction and disproportionation with the stepwise formation of Li_2_S_6_, Li_2_S_4_, Li_2_S_2_, and the end product Li_2_S.^[^ ^47^ ^]^ The Gibbs free energies of the abovementioned reactions on the Ni, CeO_2_ and Ni‐CeO_2_ heterostructure models are shown in Figure 6c, and the optimized structures of the intermediates on the Ni‐CeO_2_ heterostructure models are shown in the inset of Figure 6c. After the spontaneous conversion of S_8_ to Li_2_S_8_, the subsequent four steps for the formation of Li_2_S_6_, Li_2_S_4_, Li_2_S_2_, and Li_2_S on all models are either spontaneous (negative Gibbs free energy) or endothermic (positive Gibbs free energy). During the entire discharge process, the rate‐limiting step has the largest positive Gibbs free energy among all the steps, which is 1.21 eV for Ni, 0.67 eV for CeO_2_, and 0.38 eV for the Ni‐CeO_2_ heterostructure. The lowest Gibbs free energy of the rate‐limiting step for the Ni‐CeO_2_ heterostructure indicates that the reduction of S is thermodynamically more favorable on the Ni‐CeO_2_ heterostructure than on the Ni and CeO_2_ substrates. We calculated the density of states (DOS) of the Ni, CeO_2_ and Ni‐CeO_2_ heterostructures (Figure 6d). The Ni‐CeO_2_ heterostructure is considerably more metallic with a higher DOS at the Fermi level compared to CeO_2_. The high electrical conductivity of the Ni‐CeO_2_ heterostructure will help the LiPSs on the heterostructure surface directly gain electrons from the current collector, and enhance the rapid electrochemical reactions of S and LiPSs. The results of the abovementioned calculations effectively explain the comprehensively excellent performance of Ni‐CeO_2_‐CNF for sulfur hosts.

## 3 Conclusion

In summary, we developed a novel sulfur host material, Ni‐CeO_2_ heterostructure‐doped carbon nanofibers, that combines strong adsorption with high catalytic activity and good electrical conductivity, striking a balance between adsorption and catalytic conversion of LiPSs. The cross‐linked carbon nanofiber can also enhance electrical conductivity of the cathode and act as a second barrier to block the LiPSs shuttling. Therefore, in the presence of Ni‐CeO_2_‐CNF, the S cathode exhibits both excellent long‐term cycling stability (a low decay rate of 0.046% per cycle during 1000 cycles at 2 C) and a high‐rate capability (553.8 mAh g^−1^ at a 3 C rate). Moreover, a high reversible areal capacity of 5.3 mAh cm^−2^ can be obtained even after 50 cycles at 0.1 C when the S loading is up to 6 mg cm^−2^. The present study provides an effective strategy for accommodating the thermodynamic and kinetic characteristics of LiPS adsorption and conversion. The material prepared in this study has considerable application potential for use in high‐performance Li‐S batteries. Considering the high reversible areal capacity under high S loading, such the Li‐S batteries show great application potentials in drones, electric vehicles, portable electronics, and medical devices.

## Conflict of Interest

The authors declare no conflict of interest.

## Supporting information

Supporting Information

## Data Availability

The data that support the findings of this study are available from the corresponding author upon reasonable request.
