# Supplementary material for: Ni‐CeO2 Heterostructures in Li‐S Batteries: A Balancing Act between Adsorption and Catalytic Conversion of Polysulfide
Source: Adv Sci (Weinh). 2022 Apr 12;9(17):2105538. doi: 10.1002/advs.202105538 (PMC9189638; doi:10.1002/advs.202105538)
Supplement: Supplementary file 1 — Supporting Information [file ADVS-9-2105538-s001.pdf]

ADVANCED SCIENCE
Open Access

Supporting Information

for *Adv. Sci.*, DOI 10.1002/advs.202105538

Ni-CeO_2 Heterostructures in Li-S Batteries: A Balancing Act between Adsorption and Catalytic Conversion of Polysulfide

Yang Kong, Xin Ao, Xiao Huang, Jinglong Bai, Shangquan Zhao, Jinyong Zhang* and Bingbing Tian**

WILEY-VCH

ADVANCED SCIENCE
Open Access

Supporting Information

for *Adv. Sci.*, DOI: 10.1002/advs.202105538

Ni-CeO_2 Heterostructures in Li-S Batteries: A Balancing Act between Adsorption and Catalytic Conversion of Polysulfide

*Yang Kong, Xin Ao**, *Xiao Huang, Jinglong Bai, Shangquan Zhao, Jinyong Zhang** *and Bingbing Tian**

Supporting Information

Ni-CeO_2 Heterostructures in Li-S Batteries: A Balancing Act between Adsorption and Catalytic Conversion of Polysulfide

Yang Kong, Xin Ao*, Xiao Huang, Jinglong Bai, Shangquan Zhao, Jinyong Zhang* and Bingbing Tian*

Y. Kong, Prof. J. Zhang

School of Material and Physics, China University of Mining and Technology, Xuzhou, Jiangsu 221008, China

E-mail: jyzhang@cumt.edu.cn

Y. Kong, Dr. X. Huang, Dr. L. Bai, Prof. B. Tian

SZU-NUS Collaborative Innovation Center for Optoelectronic Science & Technology, International Collaborative Laboratory of 2D Materials for Optoelectronics Science and Technology of Ministry of Education, Institute of Microscale Optoelectronics, Shenzhen University, Shenzhen 518060, China

E-mail: tianbb2011@szu.edu.cn

Dr. S. Zhao, Dr. X. Ao

School of Materials Science and Engineering, Nanchang University, 999 Xuefu Avenue, Nanchang, Jiangxi 330031, China

E-mail: aoxin@ncu.edu.cn

Experimental Section

Synthesis of Ni-CeO_2 heterostructures doped carbon nanofiber. First, 0.1 mmol $Ni(NO_3)_2·6H_2O$ (Aladdin), 0.1 mmol $Ce(NO_3)_3·6H_2O$ (Macklin), 0.5 g polyacrylonitrile (PAN, $M_w \approx 150000$ Macklin) and 0.5 g polyvinylpyrrolidone (PVP, $M_w \approx 1300000$ Sigma) were dissolved into 10 ml N,N-dimethyl formamide (DMF, 99.5% Macklin). Then, the homogeneous sols were eventually obtained after continuous magnetic stirring for 12 h. The sols were transferred into a 5 ml syringe attached to a needle with inner diameter of 0.20 mm. The electrospinning process was performed at an applied positive voltage of 12 kV and negative voltage of 2 kV at room temperature, with the 15 cm spacing between a metal collector cathode and needle tip anode. The feeding rate was controlled as 0.045 mm min^{-1}. The humidity level was maintained around 35% RH. When the electrospinning process was finished, the nanofiber obtained on the aluminum collector. Subsequently, the as-spun nanofibers were dried at 60 ^{o}C in vacuum for 6 h. The dried electrospinning nanofibers were further stabilized in air at 210 ^{o}C (2 ^{o}C min^{-1}) for 2 h, then heated up to 900 ^{o}C (3 ^{o}C min^{-1}) for 2 h in Ar, then naturally cooling down to room temperature. The final annealed product was Ni-CeO_2 heterostructures doped carbon nanofiber (Ni-CeO_2-CNF). Besides, in order to reveal the effect of Ni-CeO_2 heterostructures, we made different samples as contrast samples. A sols contains the same amount of $Ce(NO_3)_3·6H_2O$, PAN, PVP and DMF. Another sample contains the same amount of PAN, PVP and DMF. CeO_2 carbon nanofiber (CeO_2-CNF) and carbon nanofiber (CNF) were obtained after the same experimental conditions.

Materials characterizations. The microstructure features of CNF, CeO_2-CNF and Ni-CeO_2-CNF were analyzed by using the field-emission scanning electron microscopy (SEM, Thermo APREO S) and transmission electron microscopy equipped (TEM, JEOL F200) with energy dispersive spectroscope (EDS). The Raman spectrum was measured by a Raman Spectrometer (Mstarter ScanPro) with an excitation wavelength of 532 nm. X-ray photoelectron Specific was performed to confirm the elemental composition by PHI 5000

VersaProbe III with a monochromatic Al Kα X-ray source, with a base pressure better than 5×10^{-7} for analysis. All binding energy values were referenced to the standard C 1s peak at 284.8 eV. The surface areas were determined by Brunner-Emmett-Teller (BET, ASAP 2460) method. The crystalline structure of materials was characterized by X-ray diffraction (XRD, Rigaku Ultima IV). The contents of Ni and CeO_2 were gained by inductive coupled plasma (ICP, Agilent ICPOES730). The content of sulfur in the composite was analyzed using thermogravimetric analysis (TG, TA Discover-550) from room temperature to 800 ^oC with a heating rate of 10 ^oC min^{-1} in a nitrogen atmosphere.

LiPSs adsorption tests. First, 0.2 M Li_2S_6 solution was synthesized by sublimed sulfur and Li_2S at a molar ratio of 5:1, which added to liquid mixture of 1,2-dimethoxyethane (DME) and 1,3-dioxolane (DOL) (1:1 in volume) and vigorously stirred at 80 ^oC for 12 h under argon atmosphere. The Li_2S_6 solution was diluted to 2 mM. The LiPSs adsorption tests were conducted by mixing 5 mL of Li_2S_6 solution and 25 mg of CNF, CeO_2-CNF or Ni-CeO_2-CNF. All these procedures were carried out in an Ar-filled glovebox. Then, the mixture was held at room temperature for 6 h for observing the color change. Finally, the supernatant and the solids were separated that the supernatant were used for UV-vis measurements.

Catalytic conversion of LiPSs. The symmetrical batteries were assembled with two electrodes composed of 80 wt% active material, 20 wt% PVDF and 50 μL electrolyte which was a dioxolane (DOL)/dimethoxyethane (DME) of volume ratio 1:1 containing 1.0 M LiTFSI, 0.2 M Li_2S_6. Cyclic Voltammetry measurements of the symmetrical battery were performed between -1.4 and 1.4 V at a scan rate of 1 mV s^{-1}.

Li_2S nucleation experiments. Host materials (2 mg) loaded on the carbon papers apply as work electrode. Li foil work as the counter electrode. S and Li_2S were stirred in a molar ration of 7:1 in tetraglyme solvent for 24 h to obtain the Li_2S_8 solution (0.2 mol L^{-1}). 20 μL of Li_2S_8 solution was deposited onto CNF, CeO_2-CNF and Ni-CeO_2-CNF working electrode side, and then 20 μL of LiTFSI (1.0 mol L^{-1}) without Li_2S_8 is dropped onto the Li foil. The batteries

were discharged at 0.112 mA with a voltage cut-off of 2.06 V and then kept potentiostatically at 2.05 V for Li_2S to nucleate and grow until the current fell below 10^{-5} A. The nucleation rate of Li_2S on the substrates was calculated based on Faraday's law.

Preparation of Ni-CeO_2-CNF composited sulfur. The sulfur loading was realized by the melt-diffusion strategy. Typically, 65 wt% sulfur and 35 wt% Ni-CeO_2-CNF were mixed. Then, the mixture was moved into a PTFE reactor in an Ar-filled glovebox. And it heated to 155 ^oC for 12h.

Measurement of the galvanostatic intermittent titration (GITT). A protocol of a current pulse at 0.1 C for 15 min with 15 min of rest was used. The internal resistance could be quantified by the following relation equation:

$$\Delta R_{internal}(\Omega) = \left|\Delta V_{QOCV-CCV}\right| / I_{applied}$$

where $\Delta V_{QOCV\text{-}CCV}$ is the voltage difference between the points of quasi open-circuit voltage (QOCV) and closed-circuit voltage (CCV), and I_{applied} is the applied current.

Electrochemical measurements. 80 wt% S@Ni-CeO_2-CNF composite, 10 wt% Super P conduction carbon and 10 wt% PVDF were mixed in N-methyl-2-pyrrolidone (NMP) to form a slurry. After stirring for 6 h, the slurry was coated on the aluminum foil current collector and dried at 55 ^oC overnight in a vacuum to form the working electrode. The sulfur loading density was around 1 mg cm^{-2}. The high-load sulfur electrode was prepared by using carbon paper as a base. The above slurry was coated onto the carbon paper, and placed in a vacuum drying box at 55 ^oC for 12 h, corresponding to sulfur mass loading of 3.0 - 6.0 mg cm^{-2}. All of the electrochemical measurements were tested on 2032-type coin batteries with Li foil as the anode and a Celgard 2325 membrane as the separator. The electrolyte was 1.0 M lithium bis-trifluoromethane sulfonylimide in 1,3-dioxolane (DOL) and 1,2-dimethoxyethane (DME) mixed solution (volume ration 1:1) with 1 wt % $LiNO_3$ additives. The working electrodes were cycled between 1.7 and 2.7 V vs Li/Li^+. The discharge/charge performances were characterized by the Neware battery tester (BTS5V10mA) in coin-type batteries. Specific

capacity values were calculated according to the mass of sulfur. Cyclic voltammetry (CV) between 1.7 and 2.7 V at a scan rate of 0.05 mV s^{-1} was performed using Ivium Electrochemical Workstation. Electrochemical impedance spectroscopy (EIS) was carried out from 100 kHz to 0.1 Hz with an amplitude of 10 mV on an Ivium Electrochemical Workstation.

DFT calculations. The first principle calculation was performed by using density functional theory (DFT) in the Vienna Ab initio Software Package (VASP 5.4.4) code within the Perdew–Burke–Ernzerhof (PBE) generalized gradient approximation and the projected augmented wave (PAW) method[1]. The cutoff energy for the plane-wave basis set was set to 400 eV. Monkhorst–Pack (MP) grids, which has different k-point meshes for Ni, CeO_2, and Ni-CeO_2 structure optimizations, sampled the Brillouin zone of the surface unit cell[2]. The CeO_2(111), Ni(111) and Ni-CeO_2 surfaces were determined by 3 × 3 × 1, 3 × 3 × 1, and 2 × 2 × 1 Monkhorst−Pack grid. The convergence criterion for the electronic self-consistent iteration and force was set to 10^{-5} eV and 0.01 eV/Å, respectively. The electronic structure of CeO_2 was calculated using the PBE+U approach with partly reducing the underestimation of the electronic band gap and the excessive tendency to delocalize the electron density. In this work, we set the Hubbard parameter to U − J = 4 eV for Ce, which ensures a good qualitative description of structure and electronic properties of Ce oxide. We introduced a vacuum layer of 15 Å which could avoid interactions between periodic images. The adsorption energy (E_{ads}) of the surface species is defined by

$E_{ads} = E_{total} – E_{surface} - E_{species}$,

where E_{total} represents the total energy of the adsorbed species with catalyst surface, $E_{surface}$ is the energy of the empty surface, and $E_{species}$ is the energy of the species in the gas phase.

The free energies of adsorbates and transition states at temperature T were estimated according to the harmonic approximation, and the entropy is evaluated using the following equation:

$$S(T) = k_B \sum_{i}^{ham\ DOF} \left[\frac{\varepsilon_i}{k_B T (e^{\varepsilon_i / k_B T} - 1)} - \ln(1 - e^{-\varepsilon_i / k_B T}) \right]$$

where k_B is Boltzmann's constant and DOF is the number of harmonic energies (ε_i) used in the summation denoted as the degree of freedom, which is generally 3N, where N is the number of atoms in the adsorbates or transition states. Meanwhile, the free energies of gas phase species are corrected as:

$$G_g(T) = E_{elec} + E_{ZPE} + \int C_P \, dT - TS(T)$$

where C_p is the gas phase heat capacity as a function of temperature derived from Shomate equations and the corresponding parameters in the equations were obtained from NIST.

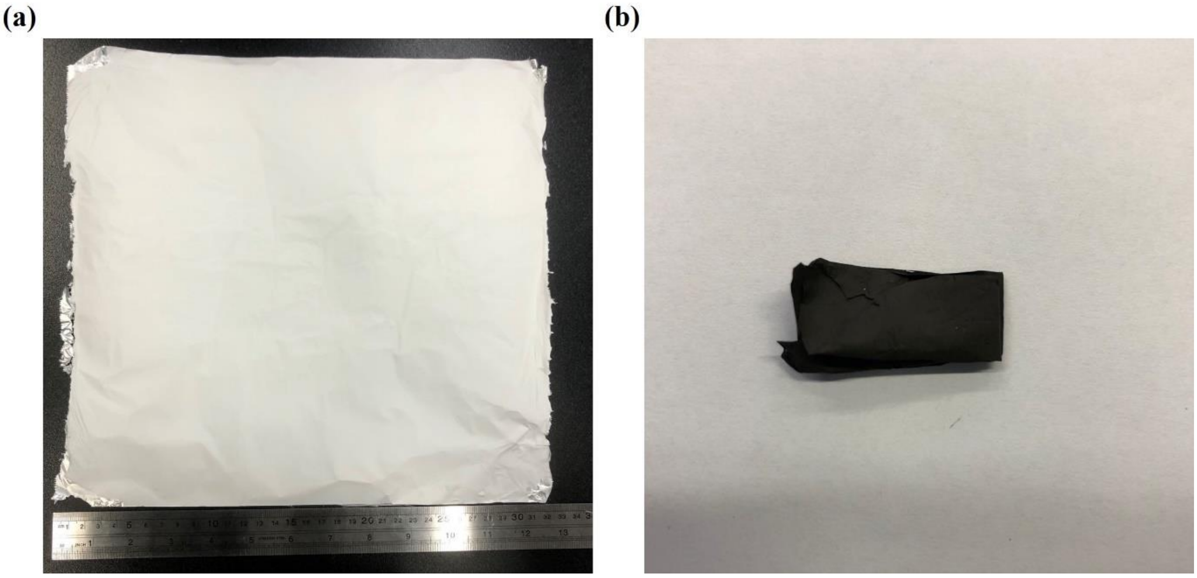

Figure S1. Optical images of (a) electrospinning and (b) annealing of Ni-CeO_2-CNF.

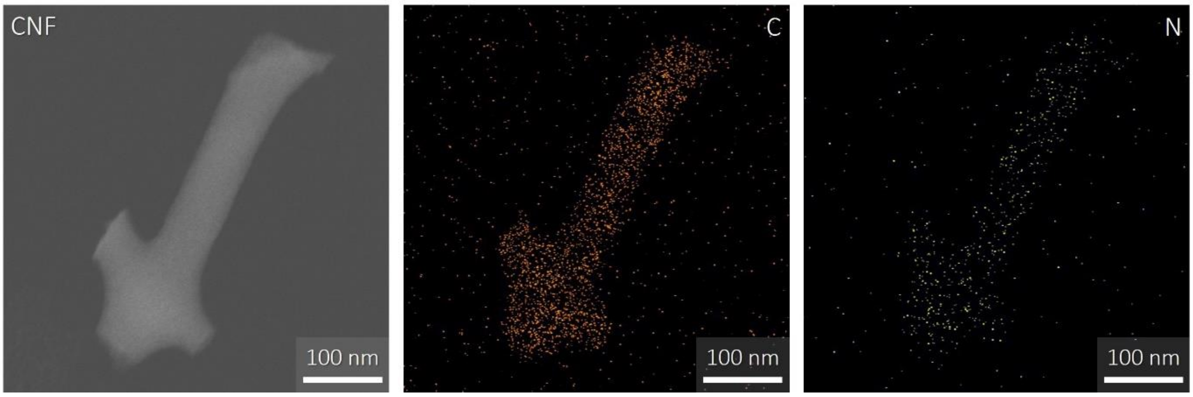

Figure S2. TEM and corresponding mapping images of CNF.

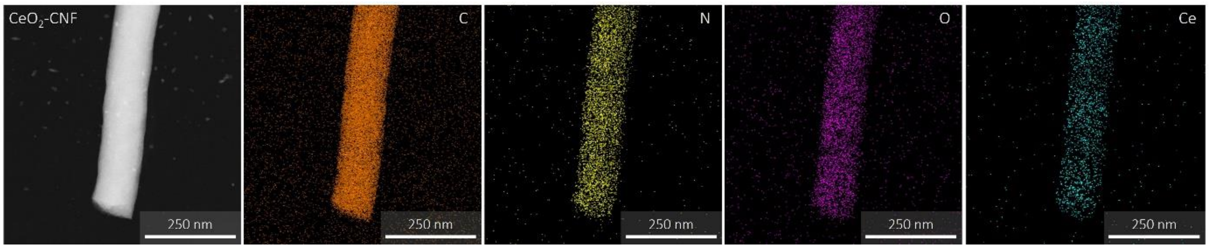

Figure S3. TEM and corresponding mapping images of CeO_2-CNF.

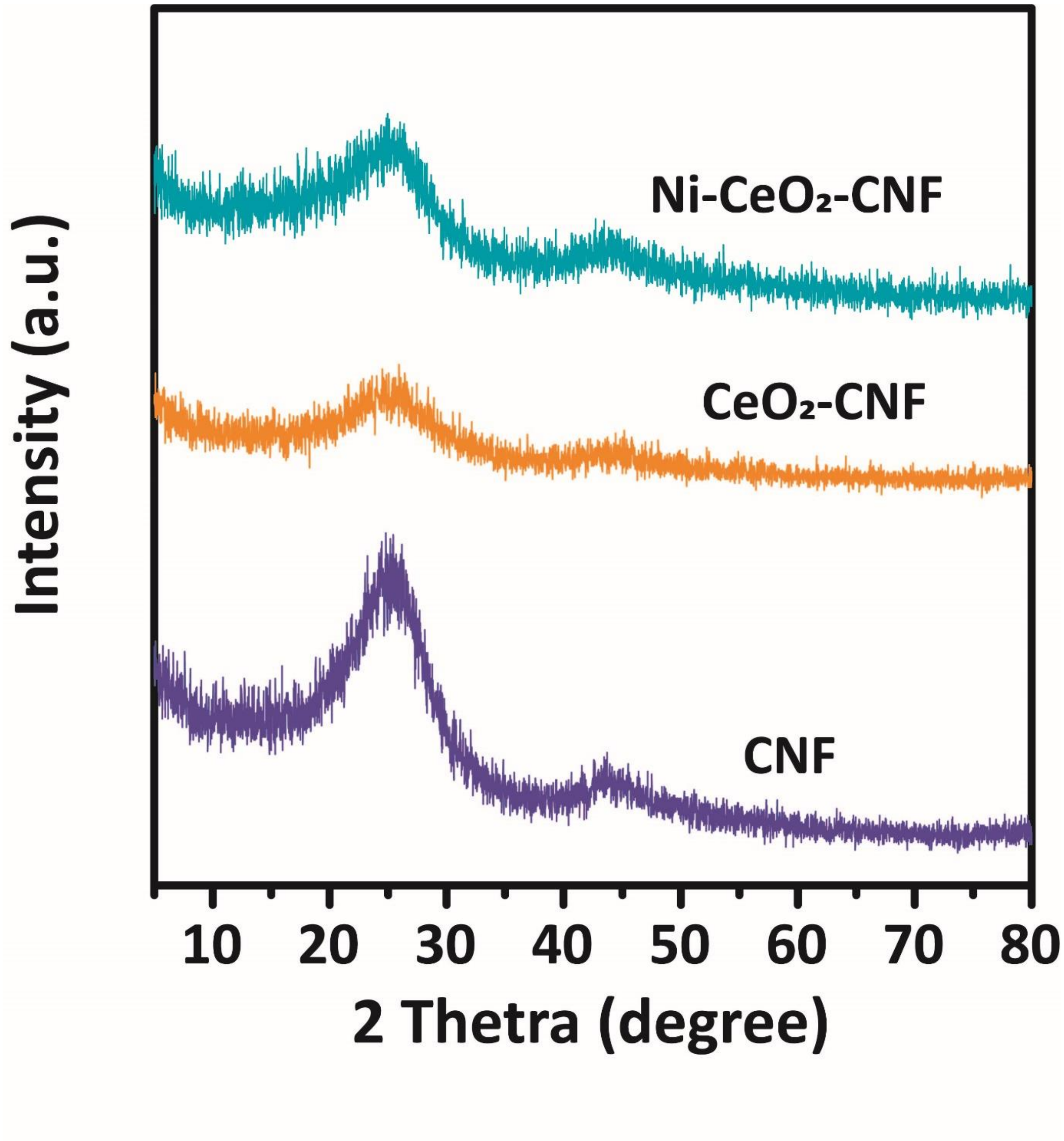

Figure S4. XRD pattern of CNF, CeO_2-CNF and Ni-CeO_2-CNF.

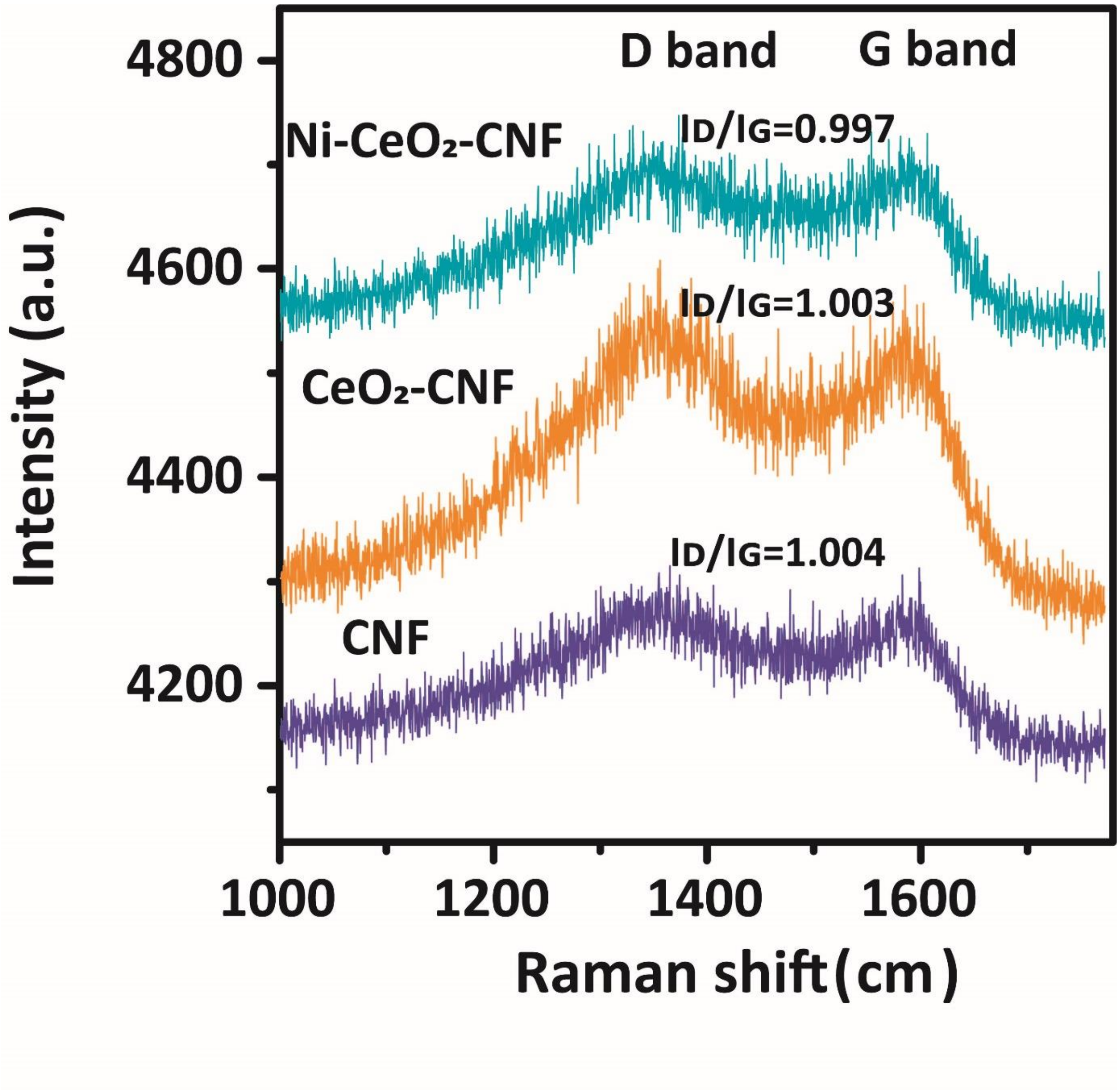

Figure S5. Raman spectra of CNF, CeO_2-CNF and Ni-CeO_2-CNF.

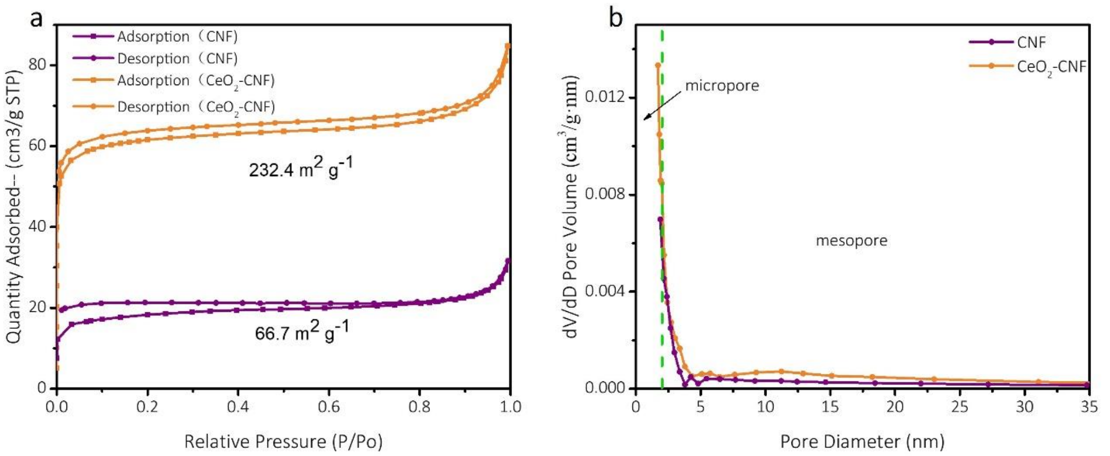

Figure S6. N_2 adsorption-desorption isotherms and the pore size distribution of CNF and CeO_2-CNF.

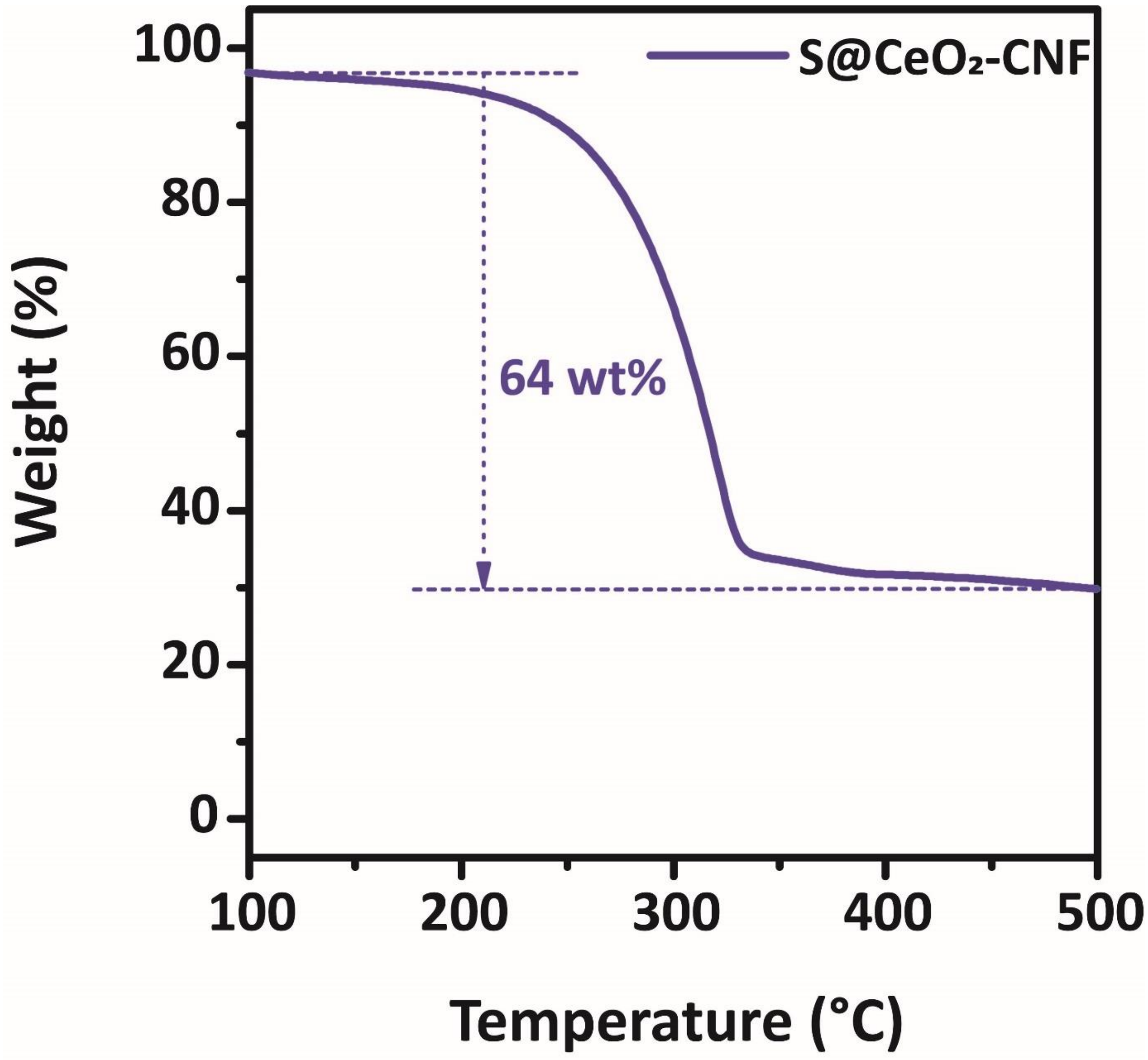

Figure S7. TGA curve of S@CeO_2-CNF.

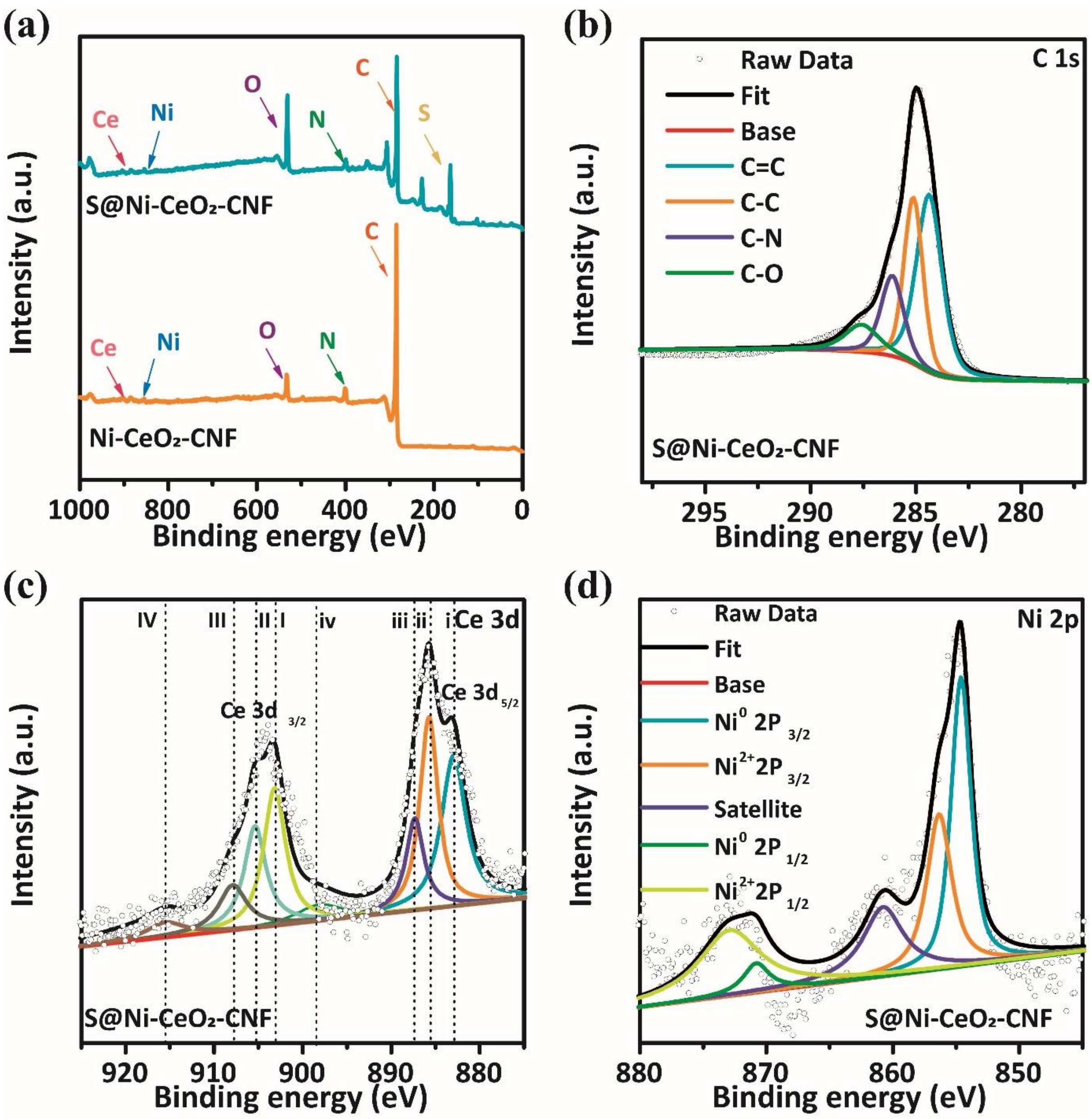

Figure S8. (a)XPS survey spectrum of Ni-CeO_2-CNF and S@Ni-CeO_2-CNF; XPS spectra of the (b)C 1s, (c)Ce 3d and (d)Ni 2p of S@Ni-CeO_2-CNF.

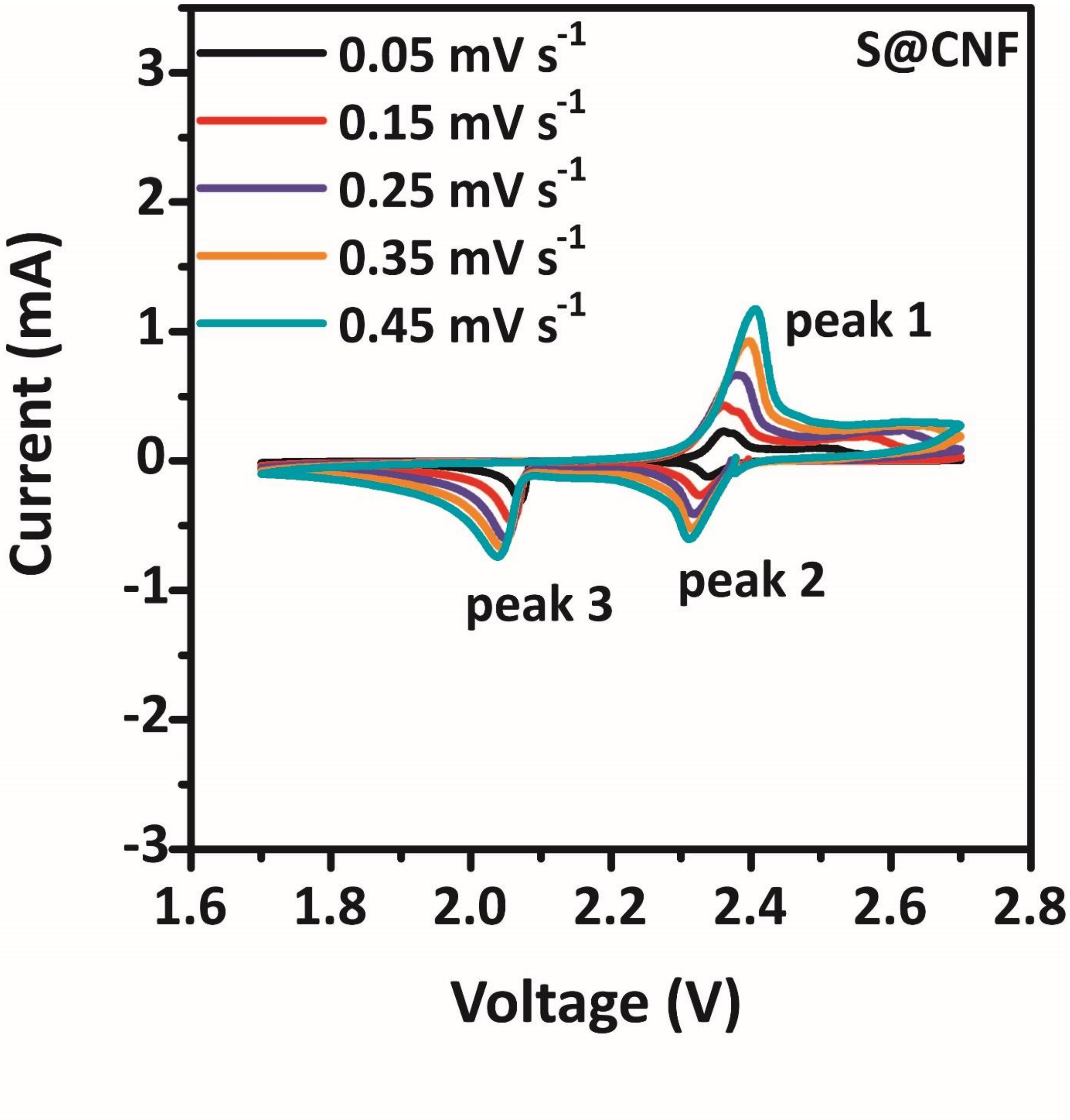

Figure S9. CV curves of S@CNF electrode at various scanning rates.

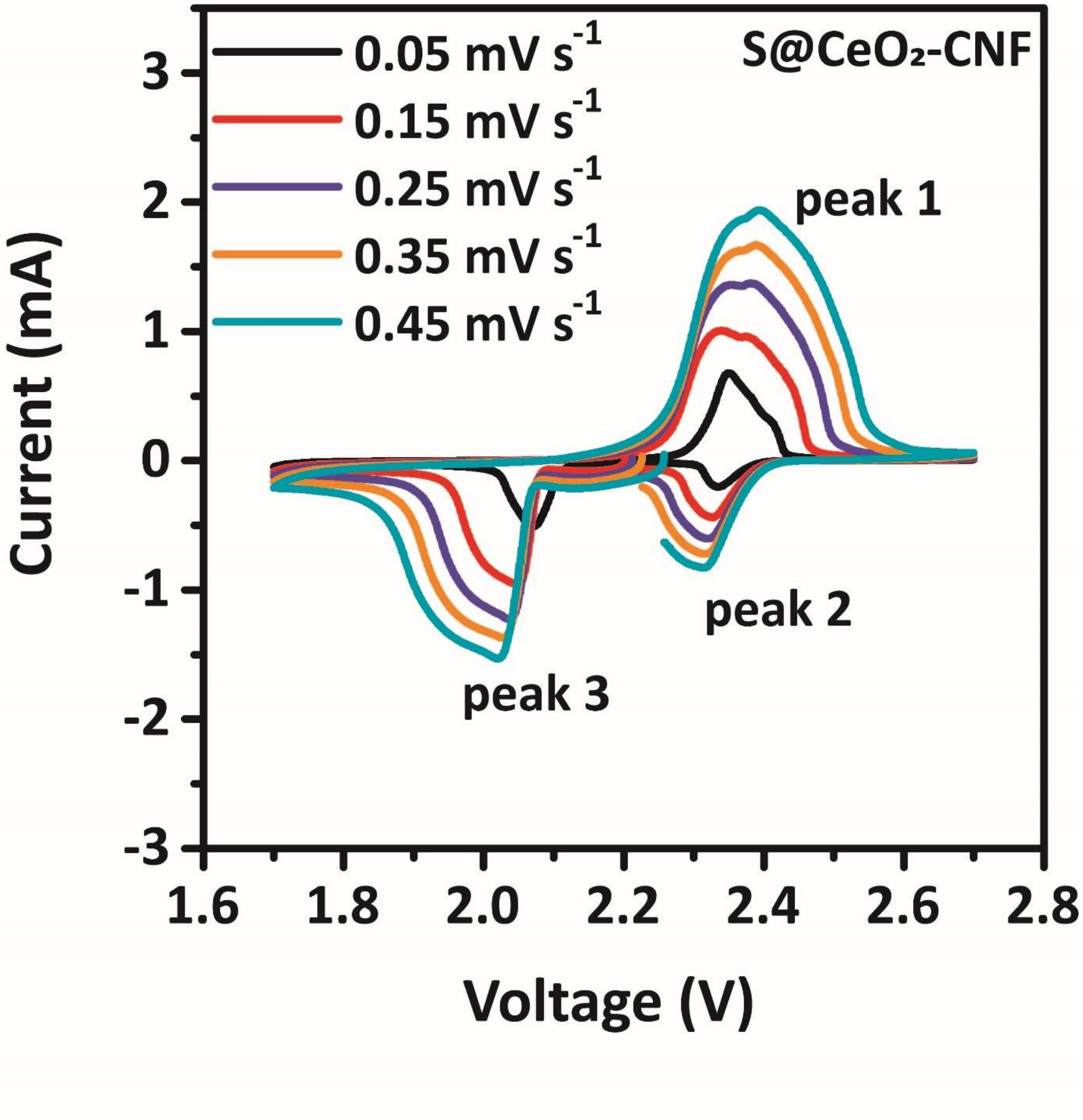

Figure S10. CV curves of S@CeO_2-CNF electrode at various scanning rates.

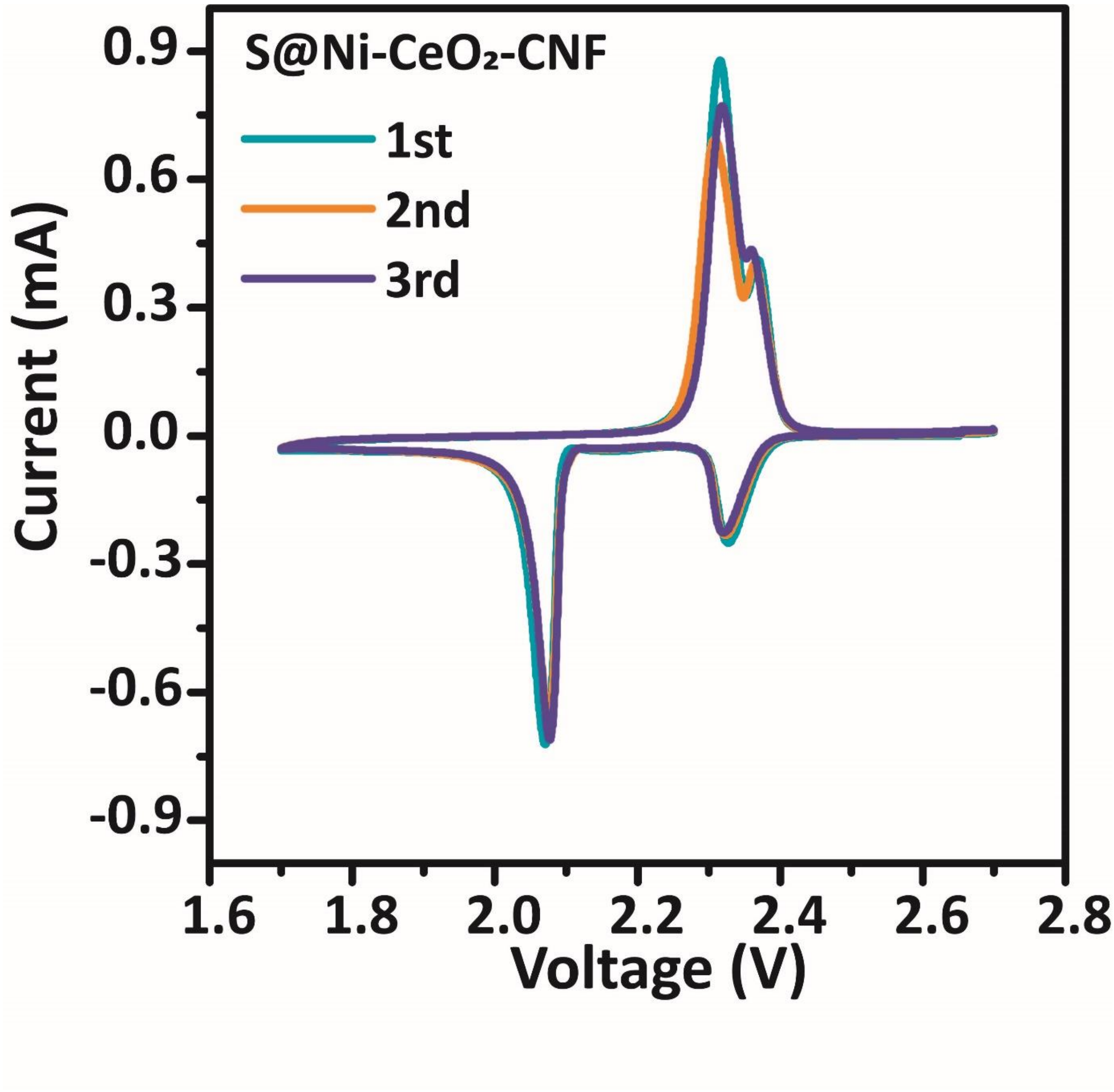

Figure S11. CV profiles of S@Ni-CeO_2-CNF cathode.

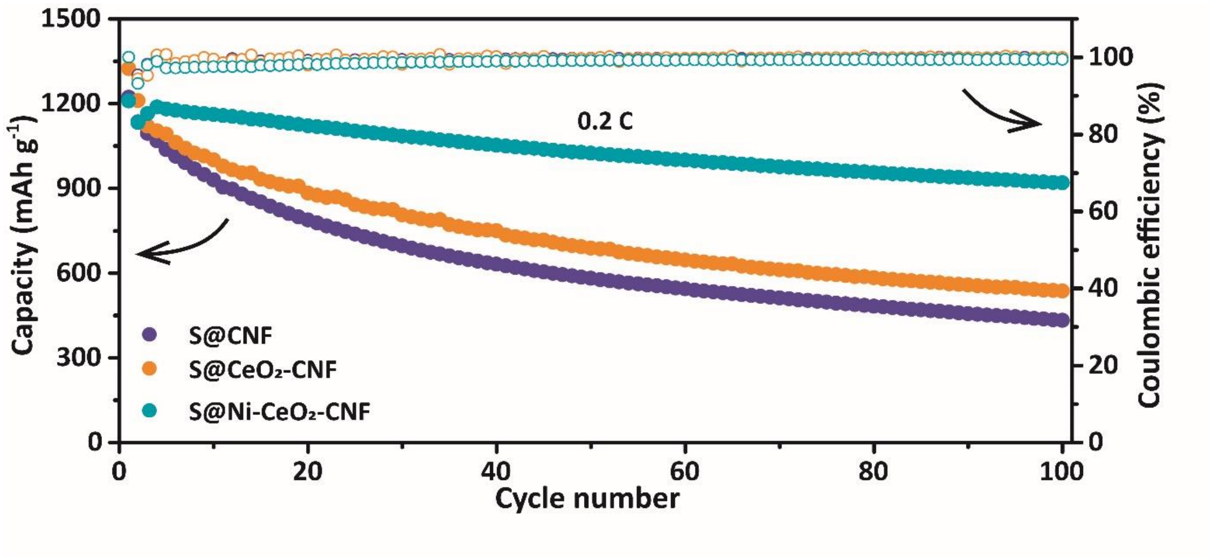

Figure S12. Cycling performance of S@CNF, S@CeO_2-CNF and S@Ni-CeO_2-CNF cathodes at 0.2 C.

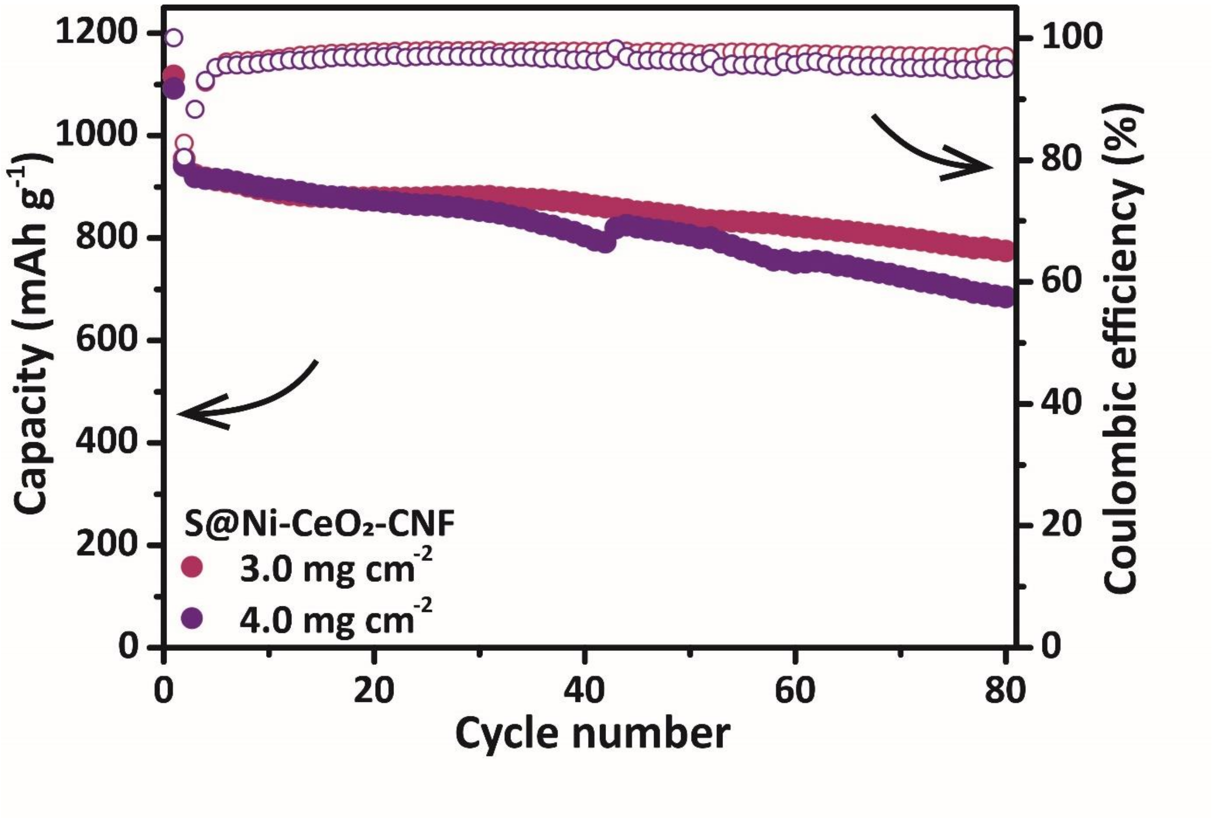

Figure S13. Cycling performance of S@Ni-CeO_2-CNF cathode at 0.1 C with high S loadings of 3.0 and 4.0 mg cm^{-2}.

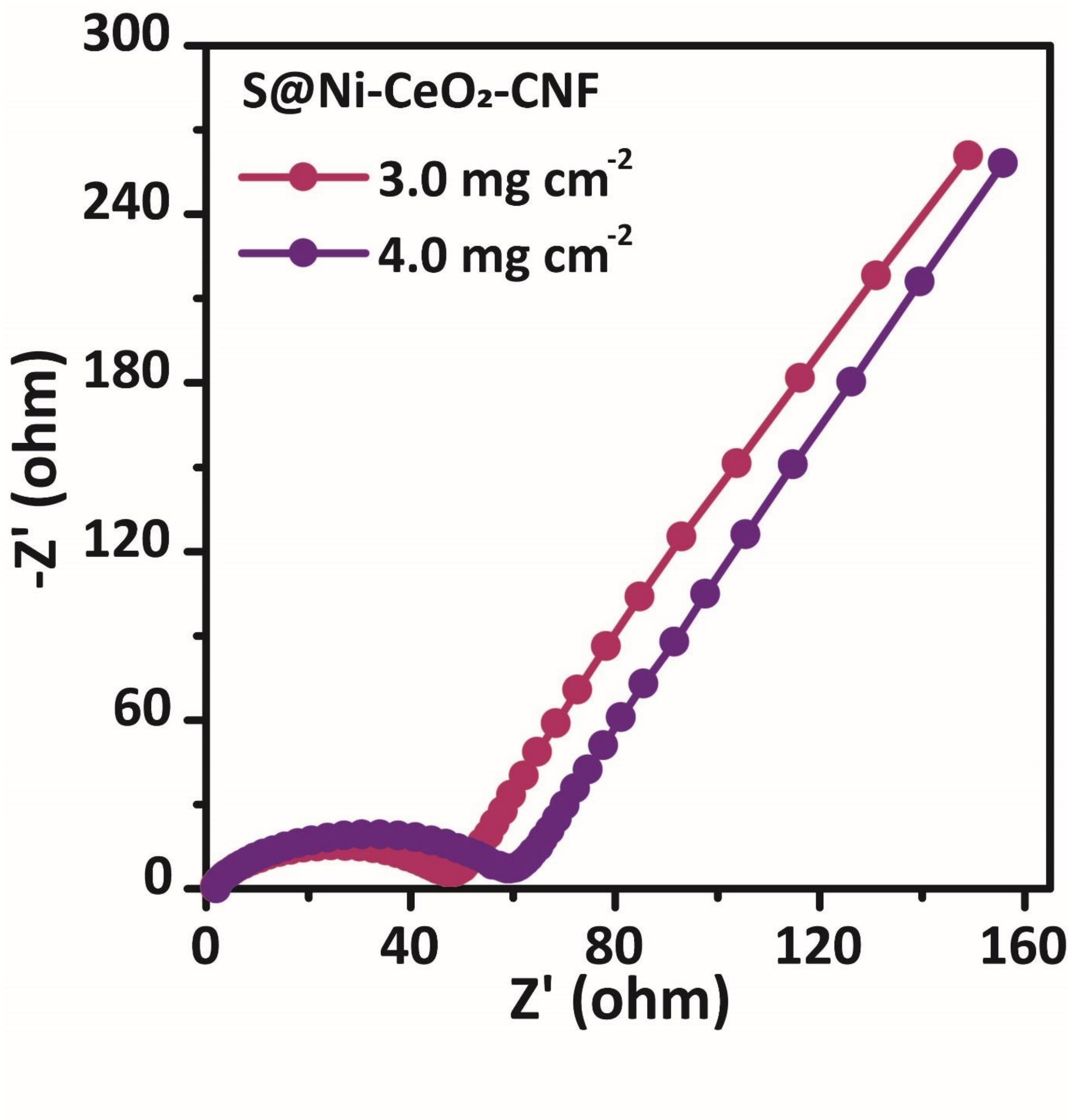

Figure S14. EIS curves of Ni-CeO_2-CNF cathode with high sulfur loading of 3.0 and 4.0 mg cm^{-2}.

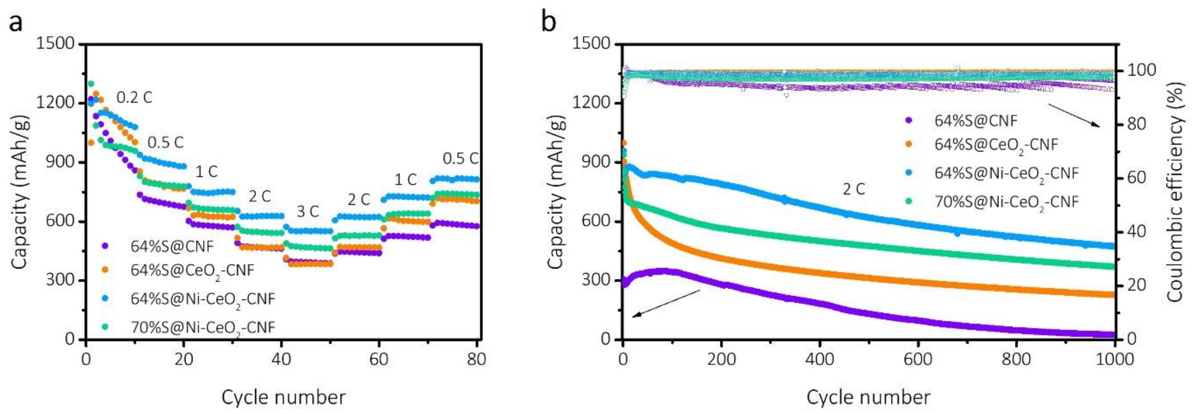

Figure S15. Electrochemical performances of the 64%S@CNF, 64%S@CeO_2-CNF, 64%S@Ni-CeO_2-CNF and 70%S@Ni-CeO_2-CNF cathodes: (a) rate performances; (b) cycling performance at 2 C.

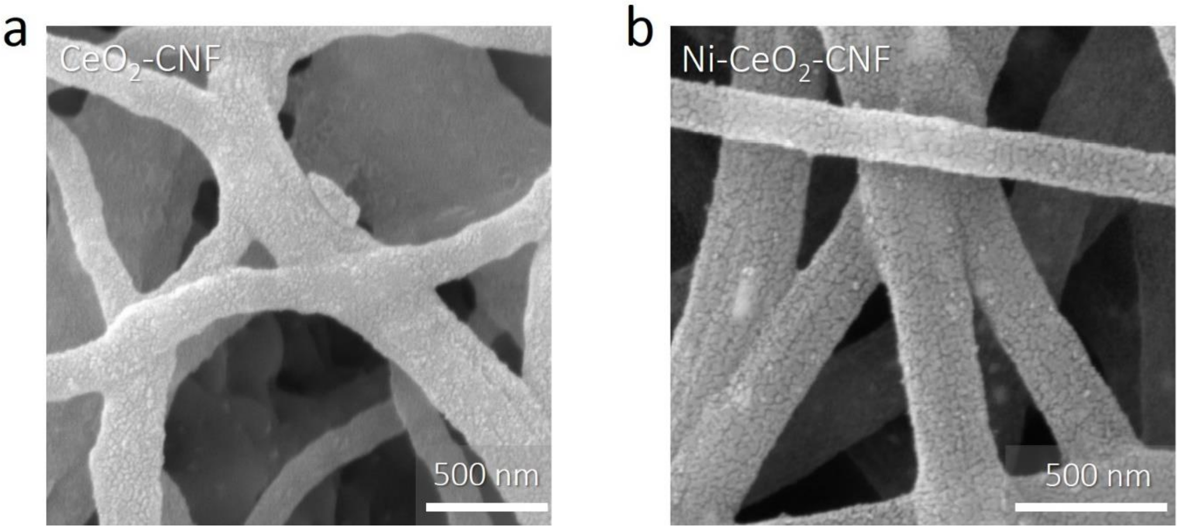

Figure S16. The higher resolution SEM of CeO_2-CNF and Ni-CeO2-CNF.

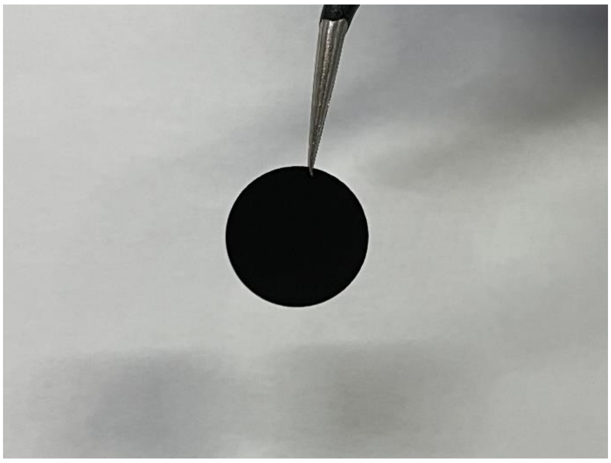

Figure S17. The physical photos of the electrodes.

Table S1. Ni and Ce weight percentage of Ni-CeO_2-CNF.

Quality/g	Volume/ml	Dilution coefficient	Element	Instrument reading	Unit	Conversion content	Unit
0.0319	25	1	Ce	0.9617	mg/L	753.7	mg/kg
0.0319	25	1	Ni	6.2291	mg/L	4881.7	mg/kg

Table S2. Impedance parameters calculated according to the equivalent circuit.

Electrodes	R_0 (Ω)	R_{ct} (Ω)	R_f (Ω)
S@Ni-CeO_2-CNF	2.5	21.2	7.2
S@CeO_2-CNF	2.0	20.1	18.5
S@CNF	4.6	29.41	24.3

Table S3. Comparison of the long-term cycling performance of Li-S batteries with recent representative work.

Materials	S loading [mg cm^{-2}]	Cycles	Current density	Decay rate [per cycle, %]	Reference
S@Ni-CeO_2-CNF	1.0	1000	2 C	0.046	This work
Ni-Co-P@C//P	1.8	1000	1 C	0.056	[3]
Ni/SiO_2/G	1.0-1.2	300	2 C	0.085	[4]
PAN/CNF-CeO_2	-	300	0.5 C	0.040	[5]
VO_2-VN	1.0	800	2 C	0.060	[6]
WS_2-WO_3	-	500	0.5 C	0.060	[7]
Mo_2C-CNOs@S	1.0	600	1 C	0.046	[8]
CeO_2/CNTP-S	-	300	2 C	0.044	[9]
CP/Fe-N-GOMC/S	3.0	500	0.5 C	0.075	[10]

Table S4. Comparison of the high sulfur loading performance of Li-S batteries with recent representative work.

Materials	S loading [mg cm^{-2}]	E/S ration [μL mg^{-1}]	Current density	InitialCapacity [mAh cm^{-2}]	cycle number	Reference
S@Ni-CeO_2-CNF	6.0	~6.67	0.1 C	7.0	50	This work
VO_2-VN	4.2	7.5	0.3 C	4.7	50	[6]
CeO_2/CNTP	4.6	-	0.5 C	5.6	100	[9]
BiOCl/G-S	4.8	-	0.2 C	5.1	50	[11]
Co@TiO_{2-x}/S	5.4	-	0.2 C	4.5	50	[12]
S/VTe_2@MgO	5.2	-	0.2 C	5.3	50	[13]
$Li_xMo_6S_8$	4.0	10	~0.15 C	~3.6	100	[14]
S@Co-N/G	6.0	-	0.2 C	~5.1	100	[15]
SVE	8.0	10	0.1 C	7.2	50	[16]

Table S5. Ce weight percentage of CeO_2-CNF.

Quality [g]	Volume [mL]	Dilution coefficient	Element	Instrument reading	Unit	Conversion content	Unit
0.0344	25	1	Ce	1.3859	mg/L	1007.2	mg/Kg

Table S6. The energy density of the Li-S batteries with three different S host materials

Rate / Electrodes	0.2C	0.5C	2.0C
S@CNF	680.4 Wh/kg	552.2 Wh/kg	166.9 Wh/kg
S@CeO_2-CNF	793.0 Wh/kg	723.5 Wh/kg	374.3 Wh/kg
S@Ni-CeO_2-CNF	1127.5 Wh/kg	906.6 Wh/kg	682.8 Wh/kg

References

[1] a) J. P. Perdew, K. Burke, M. Ernzerhof, Phys Rev Lett **1996**, *77*, 3865; b) B. Hammer, L. B. Hansen, J. K. Norskov, Phys Rev B **1999**, *59*, 7413; c) P. E. Blochl, Phys Rev B **1994**, 50, 17953; d) G. Kresse, D. Joubert, Phys Rev B **1999**, *59*, 1758.

[2] H. J. Monkhorst, J. D. Pack, Phys Rev B **1976**, *13*, 5188.

[3] Z. Wu, S. Chen, L. Wang, Q. Deng, Z. Zeng, J. Wang, S. Deng, Energy Storage Materials **2021**, *38*, 381.

[4] C. Chen, Q. Jiang, H. Xu, Y. Zhang, B. Zhang, Z. Zhang, Z. Lin, S. Zhang, Nano Energy **2020**, *76*, 105033.

[5] J. Zhang, Q. Rao, B. Jin, J. Lu, Q.-g. He, Y. Hou, Z. Li, X. Zhan, F. Chen, Q. Zhang, Chemical Engineering Journal **2020**, *388*, 124120.

[6] Y. Song, W. Zhao, L. Kong, L. Zhang, X. Zhu, Y. Shao, F. Ding, Q. Zhang, J. Sun, Z. Liu, Energy & Environmental Science **2018**, *11*, 2620.

[7] B. Zhang, C. Luo, Y. Q. Deng, Z. J. Huang, G. M. Zhou, W. Lv, Y. B. He, Y. Wan, F. Y. Kang, Q. H. Yang, Advanced Energy Materials **2020**, *10*, 2000091.

[8] G. L. Chen, Y. J. Li, W. T. Zhong, F. H. Zheng, J. H. Hu, X. H. Ji, W. Z. Liu, C. H. Yang, Z. Lin, M. L. Liu, Energy Storage Materials **2020**, *25*, 547.

[9] D. Gueon, J. Yoon, J. T. Hwang, J. H. Moon, *Chem. Eng. J.* **2020**, *390*, 124548.

[10] H. Li, D. Liu, X. Zhu, D. Qu, Z. Xie, J. Li, H. Tang, D. Zheng, D. Qu, *Nano Energy* **2020**, *73*, 104763.

[11] X. Wu, N. Liu, M. Wang, Y. Qiu, B. Guan, D. Tian, Z. Guo, L. Fan, N. Zhang, *ACS Nano* **2019**, *13*, 13109.

[12] Y. Li, X. Zhang, G. Liu, A. Gerhardt, K. Evans, A. Jia, Z. Zhang, *J. Energy Chem.* **2020**, *48*, 390.

[13] M. Wang, Y. Song, Z. Sun, Y. Shao, C. Wei, Z. Xia, Z. Tian, Z. Liu, J. Sun, *ACS Nano*

2019, *13*, 13235.

[14] Z. Yu, B. L. Wang, X. B. Liao, K. N. Zhao, Z. F. Yang, F. J. Xia, C. L. Sun, Z. Wang, C. Y. Fan, J. P. Zhang, Y. G. Wang, *Adv. Energy Mater.* **2020**, *10*, 2000907.

[15] Z. Du, X. Chen, W. Hu, C. Chuang, S. Xie, A. Hu, W. Yan, X. Kong, X. Wu, H. Ji, L. *J. Wan, J. Am. Chem. Soc.* **2019**, *141*, 3977.

[16] T. Zhang, F. Hu, W. Shao, S. Liu, H. Peng, Z. Song, C. Song, N. Li, X. Jian, *ACS Nano* **2021**, *15*, 15027.
